# Immune reactivity of *Brucella melitensis*–vaccinated rabbit serum with recombinant Omp31 and DnaK proteins

**Published:** 2013-03

**Authors:** Amir Ghasemi, Mohammad Hossein Salari, Amir Hassan Zarnani, Mohammad Reza Pourmand, Hojat Ahmadi, Abbas Mirshafiey, Mahmood Jeddi-Tehrani

**Affiliations:** 1 Department of Pathobiology, School of Public Health, Tehran University of Medical Sciences, Tehran,Iran; 2 Nanobiotechnology Research Center, Avecina Research Institue, ACECR, Tehran, Iran; 3 Immunology Research Center, Tehran University of Medical Sciences, Tehran, Iran; 4 Department of Bacterial Vaccine and Antigen Production, Pasteur Institute of Iran, Tehran, Iran; 5 Monoclonal Antibody Research Center, Avicenna Research Institute, ACECR, Tehran, Iran

**Keywords:** *Brucella*, Cloning, Immune Reactivity, ELISA, Protein Expression, Purification

## Abstract

**Background and objectives:**

*Brucella melitensis* infection is still a major health problem for human and cattle in developing countries and the Middle East.

**Materials and Methods:**

In this study, in order to screen immunogenic candidate antigens for the development of a *Brucella* subunit vaccine, a cytoplasmic protein (DnaK) and an outer membrane protein (Omp31) of *B. melitensis* were cloned, expressed in *E.coli* BL21 and then purified using Ni-NTA agarose. Immunized serum was prepared from a rabbit inoculated with attenuated *B. melitensis*.

**Results and Conclusion:**

It was proved that immunized serum contains antibodies against recombinant Omp31 (rOmp31) and DnaK (rDnaK) by Western blot and ELISA assays. The results may suggest the importance of these proteins as subunit vaccines against *B. melitensis* as well as targets for immunotherapy.

## INTRODUCTION

*Brucella* spp. are intracellular pathogens which were originally defined as facultative intracellular bacteria that preferentially infect macrophages (1, 2). Human infections with *B. melitensis* are endemic in many developing countries (3), and the incidence of brucellosis in livestock is of great economic concern due to reduced productivity, increased numbers of abortions and weak offspring, and is a major impediment to trade and export of livestock. Human brucellosis is a severe debilitating disease that requires prolonged treatment with several antibiotics, and also involves considerable medical expense, as well as loss of working hours (4). *B. melitensis Rev.1*, an attenuated smooth strain used to control *B. melitensis* infection gives heterologous protection against other *Brucella* spp. and is currently considered as the best vaccine for the prophylaxis of caprine brucellosis (5). However, major problems like the ability of this strain to cause infection in humans (6) and the development of resistance to streptomycin used to treat brucellosis, have made the health officials to prohibit its use for human vaccination (7). Therefore, a subunit vaccine that is protective against *B. melitensis* is desirable. There is an increasing interest in the study of immunogenicity and protective effects of *Brucella* outer membrane proteins (OMPs) and cytoplasmic proteins (8–10). For the first time Omp31 was cloned from *Brucella melitensis* 16M, and its predicted amino acid sequence was shown to have a significant homology (34% identity) with *Brucella* Omp25 (11). Omp31 is expressed in all *Brucella* species except in *Brucella abortus* (12). The molecular chaperone DnaK (Accession No. 1197260) belongs to the highly conserved hsp70 family, reflecting its important role in cellular metabolism (13). Induction of DnaK causes resistance to antimicrobial defense mechanisms of the macrophage in the host (14). In view of the immunological importance of the molecular chaperone DnaK and Omp31, we used purified recombinant Omp31 (rOmp31) and DnaK (rDnaK) from *B*. *melitensis* to assess the antibody response to these proteins in sera from a rabbit immunized with attenuated *B. melitensis* by ELISA and Western blot techniques.

## MATERIALS AND METHODS

### Bacterial strains and Immunization

*B. melitensis* 16M was obtained from the *Brucella* culture collection (Razi Institute,Tehran, Iran) and cultured as described (15). DNA was extracted using a DNA extraction kit (Bioneer, Daejeon, Korea).

*Escherichia coli* strain TOP10 (Invitrogen, NY, USA) was used as host for cloning experiments and for propagation of plasmids. *E. coli* strain BL21 (DE3) (Stratagene, CA, USA) was used for expression of the recombinant proteins.

A New Zealand White Rabbit was immunized intramuscularly with four doses of vaccine (10^8^ CFU of attenuated *B. melitensis Rev.1* in each dose) given 2 weeks apart. Sera were obtained before immunization and 2 weeks after the fourth dose of vaccine.

### Cloning, expression and purification of rOmp31 and rDnaK

The Gateway cloning system (Invitrogen, NY, USA) was used for cloning of a 687 bp *B*. *melitensis* DNA fragment encoding Omp31 devoid of the putative signal peptide as previously described (16, 17). The forward primers contained the cacc sequence at the 5’ end followed by the bases of the gene sequences. The primers were as follows: Sense 5'CACCATGGCCGACGTGGTTGT 3’ and f antisense 5’ GAACTTGTAGTTCAGACC 3’.

The open reading frame of DnaK consisting of 1317bp was cloned in the pET28a+ vector (Novagen, Madison, WI, USA) according to the manufacturer's instructions. The sequence information available in the *B. melitensis* genome was used to design specific primers for DnaK with *Nde*I and *Bam*HI restriction sites at the 5’ ends. The primers were as follows: sense 5’ CATATGACACCTT CTG 3 ‘, antisense 5’ GGATCCTACCGACCAGCG 3’.

*B. melitensis* genomic DNA was used as template for PCR amplification of the candidate genes using High Fidelity PCR Enzyme Mix (Fermentas, Vilnius, Lithuania). The amplified *dnak* gene from *B. melitensis* 16M was directly cloned into pTZ57R (InsTAclone™ PCR Cloning Kit) (Fermentas, Vilnius, Lithuania). Then the insert was subcloned to pET28a (+) and then transformed into *E. coli* strain TOP10 competent cells and miniprep plasmid DNA was purified from overnight cultures. The plasmid DNA of the clone containing the insert was used to transform *E. coli* strain BL21 (DE3) competent cells. Upon induction with 1 mM isopropyl-β-d-thiogalactopyranoside (IPTG) both recombinant proteins were successfully expressed in the insoluble fraction of *E. coli* cells. Purification of rDnaK and rOmp31 were done as described previously (16).

Purity was assessed by SDS-PAGE and Coomassie blue staining. Endotoxin was removed from recombinant proteins by a phase separation with Triton X-114 (18, 19). These preparations had an endotoxin content of less than 0.05 endotoxin units per mg of protein assessed by *Limulus amebocyte* lysate analysis kit (Lonza, Basel, Switzerland). The concentration of each recombinant protein was determined by Bradford method (20).

### Assessment of recombinant proteins using immunized rabbit serum: Western blot

To study the recognition of recombinant proteins by immunized rabbit serum, Western blot was used. Purified recombinant proteins were electrophoresed on a 12.5% polyacrylamide gel and transferred to a nitrocellulose membrane as mentioned above. The membrane was then incubated with immunized serum (1/2000) followed by horseradish peroxidase (HRP)-conjugated goat anti-rabbit immunoglobulin G (Avecina Research Institute, Tehran, Iran) with three washes between each step. The bound conjugates were then detected using diaminobenzidine (DAB).

### ELISA

ELISA 96-well plates (Greinerbio-one, Frickenhausen, Germany) were coated with 100 µL of 1 µg/ml and 2.5 µg/ml of rOmp31, rDnaK respectively, resuspended in 0.1 M phosphate-buffered saline (PBS) and then incubated overnight at RT. Additional wells were coated with 100 µL *B. melitensis* lysate at 1 µg/mL in PBS as positive controls.

The plates were then washed five times with PBS plus 0.05% Tween 20 (PBST) for 3 min each time. Three hundred µL of 10% fetal bovine serum (FBS) in PBS were plated and incubated for 2 h at room temperature. ELISA was then performed using 1:1000 dilutions of either normal rabbit serum or immunized rabbit serum. The plates were again washed with PBST as described earlier. One hundred µL of HRP-conjugated goat anti-rabbit immunoglobulin G (Avecina Research Institute, Tehran, Iran) (diluted 1/1000) were added to each well of the plate. The plates were again incubated for 1 h at room temperature. The plates were then washed with PBST and TMB (Pishtaz Teb, Tehran, Iran) was added to produce a color change. The reaction was stopped after 10 min by the addition of 30 µL of 20% H^2^SO^4^. An ELISA plate reader (Bio-Tek Instruments, Winooski, Vt.) was used to read the absorbance at 450-570 nm. All samples were tested in duplicates, with average absorbance values being reported.

## RESULTS

### Production of recombinant proteins

**Fig. 1:**
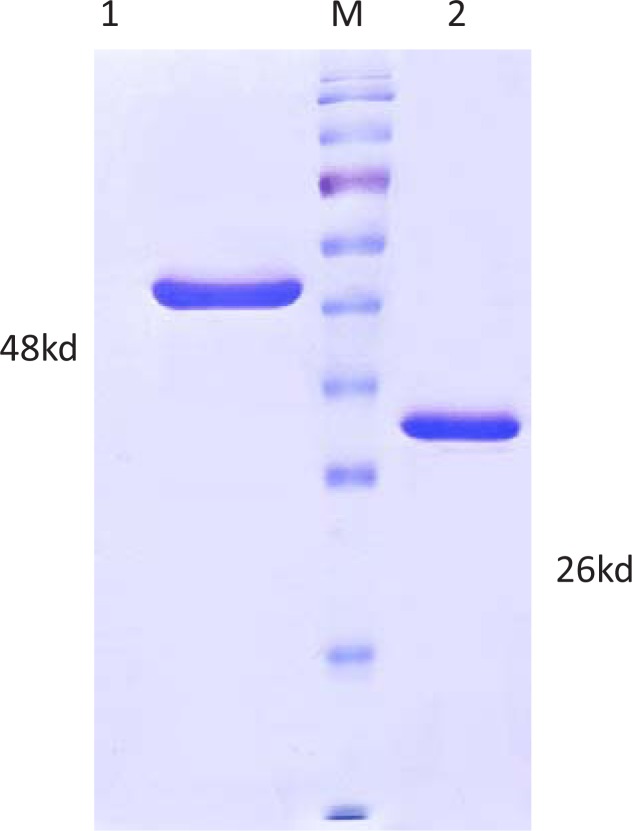
SDS - PAGE analysis of purified rOmp31 and rDNAK Proteins with Coomassie blue staining 1; Purified Recombinant DnaK Protein, 2; Purified Recombinant Omp31 Protein, M; Protein Marker (Fermentas SM 671).

Transfection pDEST-*omp*31 and pET28-*dnak* into *E.coli* BL21 (DE3) competent bacteria resulted in production of the respective proteins with the expected sizes i.e. 48 kd for DNAK and 26kd for Omp31 proteins as revealed by SDS-PAGE (Fig. 1).

### Screening of recombinant proteins with immunized rabbit serum

**Fig. 2:**
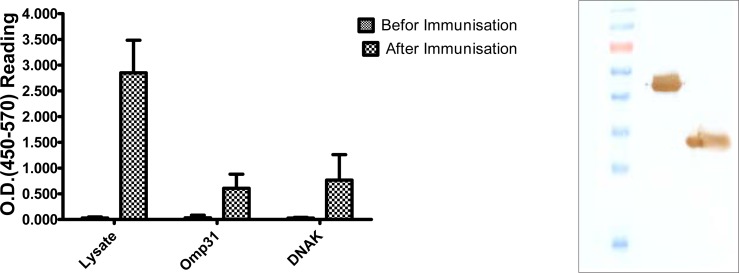
Analysis of *B. mellitensis* recombinant proteins and lysate reactivity with immunized rabbit serum. A. ELISA analysis of expressed recombinant Omp31, DnaK Protein and Lysate of *B. mellitensis* using rabbit immunized serum. B. Western blot analysis of immune reactivity of immunized rabbit serum with rOmp31 (Lane 1), rDnak (lane 2) and M; Protein Marker (Fermentas SM 671).

Immunized rabbit serum, but not pre immunized serum, strongly reacted with *B. melitensis* lysate and at a lower extent with rOmp31 and rDNAK (Fig. 2A). The two *B. melitensis* recombinant proteins reacted strongly with the immunized rabbit serum in Western blot (Fig. 2B).

## DISCUSSION

New strategies are needed to protect brucellosis while avoiding the disadvantages of the currently used live vaccines. Subunit vaccines are an attractive approach for development of effective recombinant vaccines. Although considerable work has been carried out on numerous cell surface and intracellular components, only a few antigens have shown significant protective activity (2, 15, 21, 22). The molecular chaperone (Accession No. 1197260) is named as the gene coding for DnaK protein in GenBank but it is different from the previously described *Brucella* gene that expresses the DnaK protein (23, 24). No data about the immunological properties of this antigen has been reported yet, so we decided to study the potential of DnaK protein interaction with *Brucella-*immunized rabbit serum. rDnak was cloned, expressed and purified. It showed a clear reaction with immunized rabbit serum which correlates with the hypothesis that synthesis of Hsps may occur during a stress response of the infectious organism, triggered by the hostile environment encountered during host colonization (25). It thus may be rational to propose that for a subunit vaccine against *B. melitensis* or even as a target for immunotherapy.

rOmp31, an outer membrane protein from *B. melitensis* was also cloned, expressed and purified in this study. This antigen has been shown to react with some but not all serum samples from human, dog, sheep and ram that had been infected with *Brucella* spp. (10). Moreover, rOmp31 has also been elegantly shown to react with human positive pooled serum (26). In addition, immunization of animals with *B. ovis* encoded rOmp31, alone or together with R-LPS type *B.ovis*, was reported to have developed an acceptable protection against *B.ovis* infection in the immunized mice (16). Analysis of rOmp31 interaction with immunized rabbit serum in the present study showed that rOmp31 could react much more strongly in ELISA than pre-immunized rabbit serum. These data may also suggest rOmp31 as a good candidate for subunit vaccine against *B. melitensis*.
